# *Plant* Polyphenols Attenuate DSS-induced Ulcerative Colitis in Mice via Antioxidation, Anti-inflammation and Microbiota Regulation

**DOI:** 10.3390/ijms241310828

**Published:** 2023-06-29

**Authors:** Huan Chen, Ying Li, Jinrui Wang, Tingting Zheng, Chenyang Wu, Mengyao Cui, Yifan Feng, Hanyi Ye, Zhengqi Dong, Yunjie Dang

**Affiliations:** 1 Drug Delivery Research Center, Institute of Medicinal Plant Development, Chinese Academy of Medical Sciences, Peking Union Medical College, Beijing 100193, China; 15030413919@163.com (H.C.); yli@implad.ac.cn (Y.L.); 15776622452@139.com (J.W.); t408956066@163.com (T.Z.); wuchenyang54@163.com (C.W.); cuimengyao5521@163.com (M.C.); fyf5501@163.com (Y.F.); yehy6620@163.com (H.Y.); 2 Department of Pharmaceutics, School of Pharmaceutical Sciences, Hebei Medical University, Shijiazhuang 050017, China; 3 Key Laboratory of Bioactive Substances and Resources Utilization of Chinese Herbal Medicine, Ministry of Education, Chinese Academy of Medical Sciences, Peking Union Medical College, Beijing 100094, China; 4 Key Laboratory of New Drug Discovery Based on Classic Chinese Medicine Prescription, Beijing 100700, China; 5 Beijing Key Laboratory of Innovative Drug Discovery of Traditional Chinese Medicine (Natural Medicine) and Translational Medicine, Beijing 100700, China

**Keywords:** polyphenols, antioxidants, ulcerative colitis, gut microbiota, anti-inflammation

## Abstract

The pathogenesis of ulcerative colitis (UC) is associated with inflammation, oxidative stress, and gut microbiota imbalance. Although most researchers have demonstrated the antioxidant bioactivity of the phenolic compounds in plants, their UC-curing ability and underlying mechanisms still need to be further and adequately explored. Herein, we studied the antioxidation–structure relationship of several common polyphenols in plants including gallic acid, proanthocyanidin, ellagic acid, and tannic acid. Furthermore, the in vivo effects of the plant polyphenols on C57BL/6 mice with dextran-sulfate-sodium-induced UC were evaluated and the action mechanisms were explored. Moreover, the interplay of several mechanisms was determined. The higher the number of phenolic hydroxyl groups, the stronger the antioxidant activity. All polyphenols markedly ameliorated the symptoms and pathological progression of UC in mice. Furthermore, inflammatory cytokine levels were decreased and the intestinal barrier was repaired. The process was regulated by the antioxidant-signaling pathway of nuclear-erythroid 2-related factor 2. Moreover, the diversity of the intestinal microbiota, Firmicutes-to-Bacteroides ratio, and relative abundance of beneficial bacteria were increased. An interplay was observed between microbiota regulation and oxidative stress, immunity, and inflammatory response. Furthermore, intestinal barrier repair was found to be correlated with inflammatory responses. Our study results can form a basis for comprehensively developing plant-polyphenol-related medicinal products.

## 1. Introduction

Ulcerative colitis (UC) is a common non-specific inflammatory bowel disease (IBD) characterized by chronic inflammation and ulceration of the rectal and colon mucosa. Clinical symptoms include pain, weight loss, blood in the stool, and intestinal mucosal ulcers [1,2,3,4,5]. At present, there is still no cure available for UC in clinical settings. Furthermore, recurrence causes immense economic and mental distress to patients and their families. Therefore, it is extremely important to study UC pathogenesis and identify effective drugs to treat this condition. UC pathogenesis includes inflammation, to the colon barrier damage, and oxidative stress imbalance and the intestinal microbial environment imbalance [1].

Oxidative stress is critical in UC pathogenesis. Organisms continuously produce reactive nitrogen (RNS) and reactive oxygen species (ROS) via mitochondrial bioenergetics and oxidative metabolism, and excessive production of ROS will lead to oxidative damage [6]. On the other hand, the disruption of the microbial barrier, including an imbalance in the intestinal microbial environment, decreased bacterial diversity [7], and excessive proliferation of Gram-negative bacteria, can lead to the production of large amounts of lipopolysaccharides (LPSs), resulting in an excessive production of ROS or RNS [8]. Excess ROS/RNS affects colon epithelial cells and immune cells, thereby disrupting the immune barrier and exacerbating the inflammatory response [9,10]. Intestinal microbiota disorders are characterized by an imbalance between beneficial and pathogenic bacteria. Beneficial bacteria play immune barrier roles by regulating host immune cells, whereas some pathogenic bacteria induce inflammatory cytokines via immune cell interactions or their metabolites, aggravating intestinal damage [11]. The mechanical barrier of the intestinal tract mainly includes tight junction (TJ) proteins. TJ proteins are essential for maintaining gut homeostasis [12]. TJ proteins are responsible for regulating water, electrolyte, and nutrient uptake by the cells [13]. Destruction of the mechanical barrier of the intestinal tract will cause bacteria and antigens in the intestinal cavity to enter the intestinal mucosa lamina propria, aggravating the intestinal damage. Intestinal antigens translocate to mucosal lamina propria and activate lamina propria immune cells, producing a significant amount of inflammatory cytokines and inflammatory mediators [14]. Therefore, UC pathogenesis is a complex process, with close involvement of the mechanical, immune, and microbial barriers of the intestinal tract. Therefore, treatment strategies for UC should consider the regulation of multiple mechanisms.

Polyphenols are widely found in tea, fruits, and medicinal plants [15,16,17], including chlorogenic acid, hydrolytic tannins, and flavonoids [18]. Gallic acid (GA), as an active component, has been found in many plants [19]; proanthocyanidins (PC), known as condensed tannins, are widely available in the bark of many plants [20]; ellagic acid (EA) is a natural polyphenol found in many fruits and vegetables [21,22]. Tannic acid (TA) is found mainly in the seeds of legumes and usually acts as a defense against oxidative damage to the seeds [23]. A study has reported that polyphenols exhibit excellent antioxidant and anti-inflammatory activities [24]. Moreover, the antioxidant activity of polyphenols depends largely on the presence of structural groups and the number of hydroxyl groups in a molecule [25]. Furthermore, polyphenols are sensitive to some intestinal pathogenic bacteria and can affect host immune response by causing changes in microbiota. The long-term consumption of foods rich in polyphenols can reduce the risk of various diseases [26,27], including the prevention and relief of IBD [19]. Most researchers have made efforts to elucidate the antioxidant bioactivity of the plant phenolic compounds [28,29,30]; only a few have adequately explored phenolic compounds as agents to cure UC and elucidated the relationship of between antioxidation and gut microbiota regulation.

**Figure 1:**
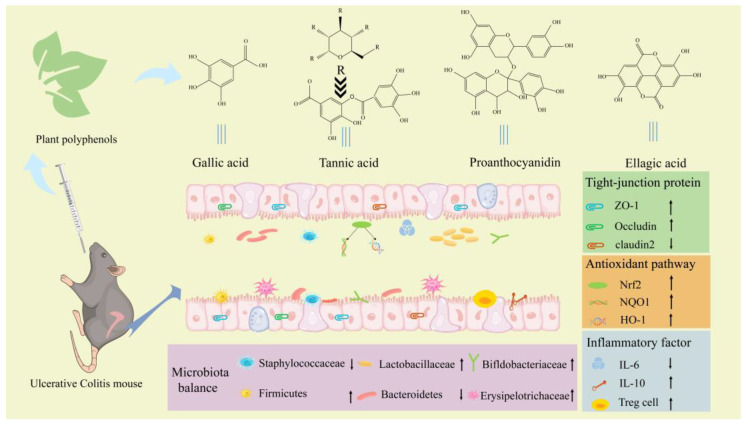
The potential mechanism of polyphenols in plants in alleviating ulcerative colitis (UC). Nuclear-factor-erythroid 2–related factor 2 (Nrf2); heme oxidase-1 (HO-1); NAD(P)H dehydrogenase [quinone] 1 (NQO1); interleukin-6 (IL-6); and interleukin-10 (IL-10).

Therefore, the present study aimed to elucidate the antioxidant effects of polyphenols in plants including GA, PC, EA and TA and the structure–function relationship, and to explore the mechanism of relieving UC, including alleviating oxidative stress, intestinal barrier repair, and intestinal microbiota regulation (Figure 1). Simultaneously, the relationship between microbiota imbalance and oxidative stress, microbiota and immune cells, and inflammatory cytokines in intestinal barrier was elucidated.

## 2. Results

### 2.1. Antioxidant Performance

**Figure 2:**
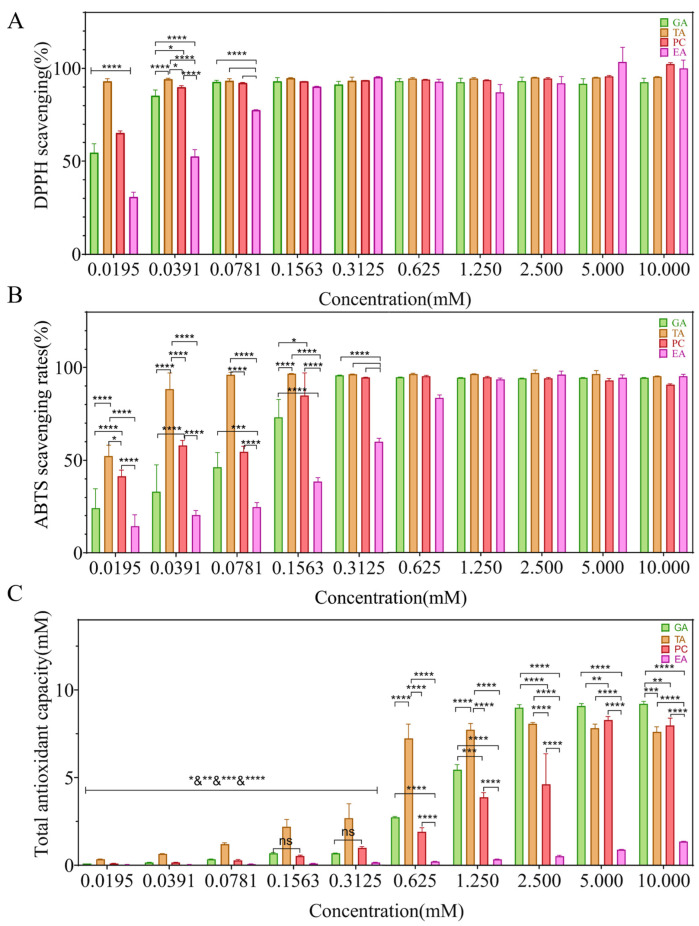
Antioxidant performance of the four polyphenols. (**A**) The DPPH-scavenging efficiency of the polyphenols; (**B**) the ABTS-scavenging efficiency of the polyphenols; (**C**) the total antioxidant capacity of the polyphenols. 1,1-diphenyl-2-picrylhydrazyl (DPPH·) and 2, 2′-azino-bis (3-ethylbenzothiazoline-6-sulfonic acid) (ABTS), gallic acid (GA), proanthocyanidins (PC), ellagic acid (EA) and tannic acid (TA), ^ns^—no significance, * *p* < 0.05, ** *p* < 0.005, *** *p* < 0.0005, **** *p* < 0.0001.

The antioxidant activity of the polyphenols was evaluated by measuring the scavenging ability of 1,1-diphenyl-2-picrylhydrazyl (DPPH·) and 2, 2′-azino-bis (3-ethylbenzothiazoline-6-sulfonic acid) (ABTS) free radicals and the total antioxidant capacity. Figure 2 presents the results. The DPPH· radical scavenging rate of four polyphenols at the concentration of 10–0.1563 mM was >90%. The DPPH· radical scavenging rate of GA, TA and PC were significantly higher than EA at the concentration of 0.078 mM. The DPPH· radical scavenging rates of TA were significantly better than those of the other three groups, GA and PC were better than EA, and PC was better than GA at the concentration of 0.039 mM. The scavenging ability of DPPH· radical was TA > PC > GA and EA at the concentration of 0.0195 mM (Figure 2A).

As shown in Figure 2B, the ABTS scavenging rates of four polyphenols were >80% even at concentrations of 10–0.625 mM. The ABTS scavenging rates of GA, TA, and PC were significantly higher than that of EA at 0.3125 mM. With a decrease in concentration (0.1563 mM~0.0195 mM), the ABTS scavenging ability of the four polyphenols exhibited a general decreasing trend. The ABTS scavenging rate of TA was significantly higher than that of the other compounds, followed by PC, GA and EA.

As shown in Figure 2C, the concentration of ferrous sulfate standard solution was used to indicate the total antioxidant capacity. All four compounds exhibited antioxidant activity. The total antioxidant capacity of EA was the weakest at a concentration of 10–0.0195 mM. The total antioxidant capacity of four polyphenols exhibited a decreasing trend as the concentrations decreased (i.e., 2.5–0.0195 mM).

### 2.2. In Vivo Assessment of the Restorative Effects of UC

#### 2.2.1. Polyphenols Alleviate Dextran-Sulfate-Sodium-Salt (DSS)-Induced Colitis

**Figure 3:**
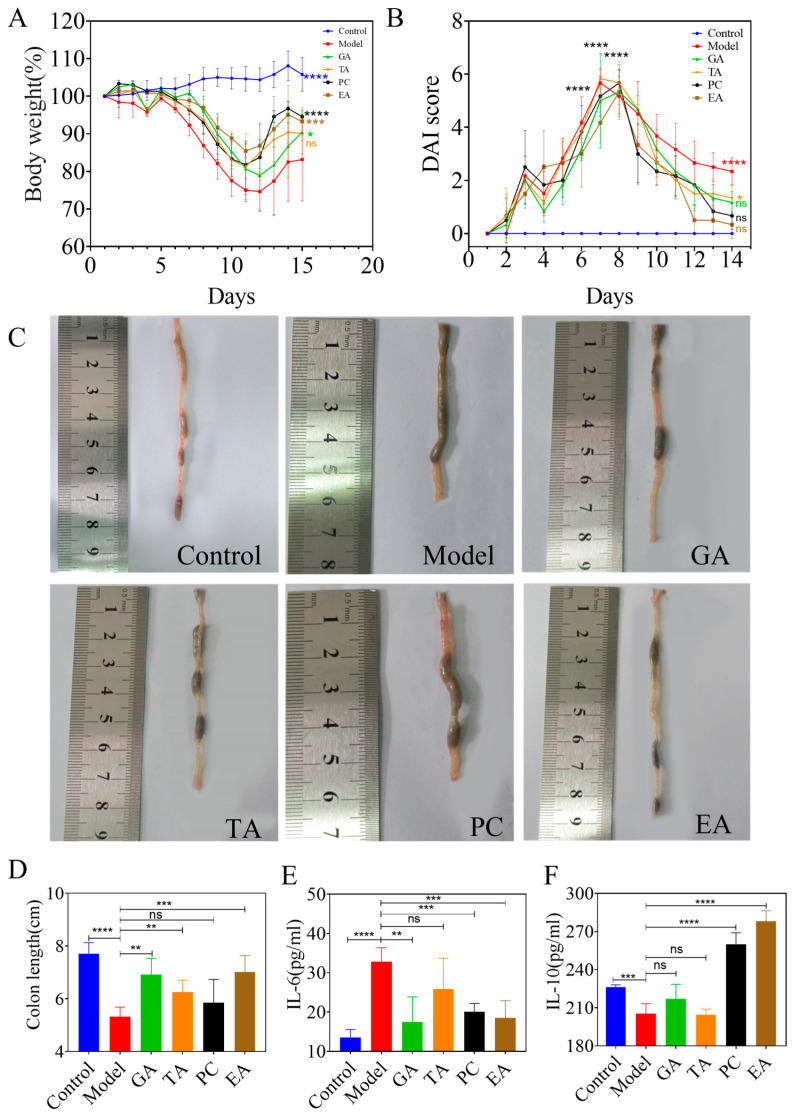
Polyphenols alleviate the symptoms of UC in mice (*n* = 6 per group). (**A**) The rate of weight change in mice; (**B**) the disease activity index (DAI) score; (**C**) representative pictures of the colon and (**D**) average colon length; (**E**) measurement of the level of IL-6 and (**F**) IL-10 in the serum with an ELISA kit. Gallic acid (GA); proanthocyanidins (PC); ellagic acid (EA); and tannic acid (TA), ^ns^—no significance, * *p* < 0.05, ** *p* < 0.005, *** *p* < 0.0005, **** *p* < 0.0001.

To determine whether polyphenols can ameliorate colon injury and inflammation, we assessed the effect of the polyphenols on 3% DSS-induced UC mice. In the established model, the weight of the mice decreased in the DSS group. After administration, the weight of the mice in all groups increased in varying degrees (Figure 3A), the weight gain in the PC group, EA group and GA group were higher than that of in the model group. Disease activity index (DAI) score of the PC, EA, and GA groups was lower than that of the model group (Figure 3B). Figure 3C,D demonstrate that GA, EA, TA alleviate colon shortening. Furthermore, serum IL-6 and IL-10 levels were detected using enzyme-linked immunosorbent assay; GA, EA, and PC significantly decreased IL-6 levels (Figure 3E) and PC, EA increased IL-10 levels (Figure 3F).

#### 2.2.2. Effects of Polyphenols on Colon Histopathology

**Figure 4:**
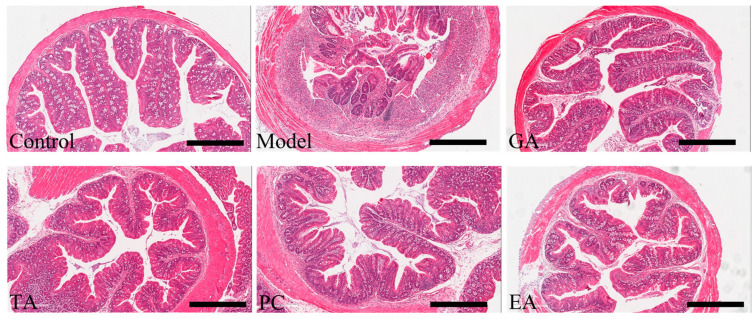
Pathological examination of mouse colon tissue (×200) (*n* = 6 per group), scale bar = 500 μm. Gallic acid (GA); proanthocyanidins (PC); ellagic acid (EA); and tannic acid (TA).

Hematoxylin-eosin (H&E) staining results in the colon revealed distortion of the crypt architecture and hyperplasia, mucosal epithelial cell degeneration/necrosis, and large amounts of inflammatory cell infiltration in the model group (Figure 4). In the treatment groups, the epithelial cells were mild or non-degenerative/necrotic and a small number of inflammatory cells were present. Among them, the histological characteristics of the GA and TA groups were similar to those of the healthy control group.

#### 2.2.3. Effects of Polyphenols on Regulatory T (Treg) Cell Expression

**Figure 5:**
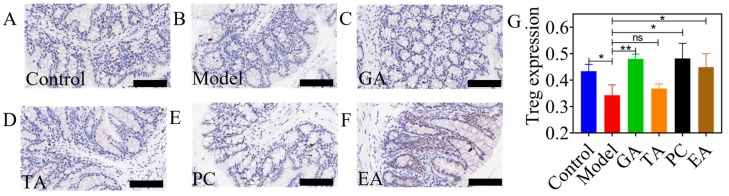
Effects of polyphenols on regulatory T cells in mice with UC (*n* = 6 per group). (**A**–**F**) Immunohistochemical staining of regulatory T cells; (**G**) semi-quantitative results of regulatory T cells levels, scale bar = 100 μm; gallic acid (GA); proanthocyanidins (PC); ellagic acid; (EA) and tannic acid (TA); ^ns^—no significance; * represents *p* < 0.05; ** represents *p* < 0.005.

The expression of Treg cells was detected using immunohistochemical (IHC)-staining-labeled FOXP3 antigen. The results are shown in Figure 5. Brown particles represent positive staining results, and semi-quantitative analysis was performed on them (Figure 5G). Treg cell expression in the model group was significantly decreased compared with the control group. Furthermore, Treg cell expression was significantly increased in the GA, PC, and EA groups when compared with the model group, implying that GA, PC, and EA could reverse the decreased expression of Treg cells in ulcerative colitis.

#### 2.2.4. Effect of Polyphenols on the Expression of Tight Junction

**Figure 6:**
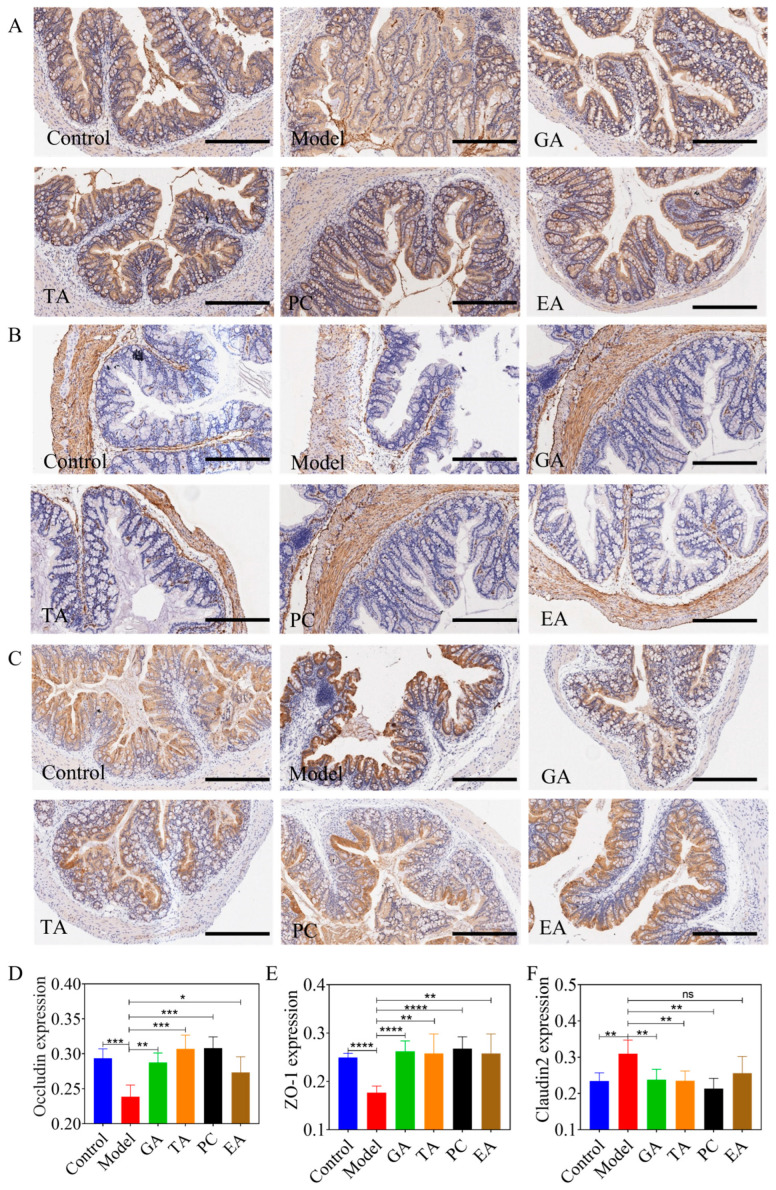
Effects of polyphenols on intestinal tight junction in mice with UC (*n* = 6 per group). (**A**) Immunohistochemical staining of occludin (×400), scale bar = 300 μm; (**B**) immunohistochemical staining of ZO-1 (×400), scale bar = 300 μm; (**C**) immunohistochemical staining of claudin-2 (×400), scale bar = 300 μm; (**D**) semi-quantitative results of occludin levels; (**E**) semi-quantitative results of ZO-1 levels; (**F**) semi-quantitative results of cluadin-2 levels; gallic acid (GA); proanthocyanidins (PC); ellagic acid (EA); and tannic acid (TA); ^ns^—no significance; * represents *p* < 0.05; ** represents *p* < 0.005; *** represents *p* < 0.0005; **** represents *p* < 0.0001.

The intact intestinal epithelial TJ plays an essential role in preventing inflammatory reactions [31]. IHC staining was performed to elucidate the levels of the TJ proteins, zonula occludens 1 (ZO-1), claudin-2, and occludin. The results are presented in Figure 6. Compared with the control group, occludin levels in the model group were significantly decreased (Figure 6A,D). Furthermore, occludin levels were significantly increased in the treatment groups compared to the model group. Compared with the control group, ZO-1 levels were significantly decreased in the model group (Figure 6B,E). Furthermore, ZO-1 levels in the administration groups were significantly increased compared the model group. Compared with the control group, claudin-2 levels were significantly increased in the model group (Figure 6C,F). Moreover, claudin-2 levels were significantly decreased in the GA, TA, and PC groups compared with the model group. The increased claudin-2 level was not improved in the EA group.

#### 2.2.5. Antioxidant Mechanism of Polyphenols

**Figure 7:**
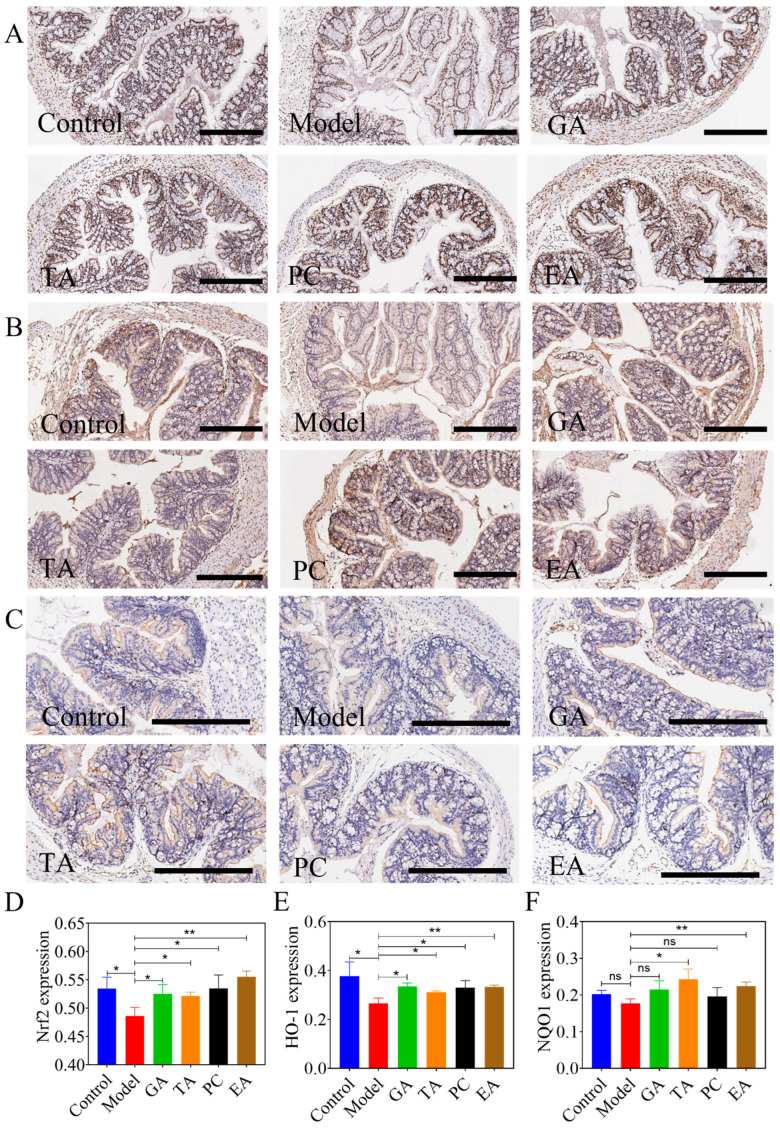
Effect of polyphenols on antioxidant pathways in mice with UC (*n* = 6 per group). (**A**) Immunohistochemical staining of Nrf2 (×400), scale bar = 300 μm; (**B**) immunohistochemical staining of HO-1 (×400), scale bar = 300 μm; (**C**) immunohistochemical staining of NQO1 (×600), scale bar = 300 μm; (**D**) average optical density (AOD) of semi-quantitative results of Nrf2 levels; (**E**) AOD of semi-quantitative results of HO-1 levels; (**F**) AOD of semi-quantitative results of NQO1 levels; gallic acid (GA); proanthocyanidins (PC); ellagic acid (EA); and tannic acid (TA); ^ns^—no significance; * represents *p* < 0.05; ** represents *p* < 0.005.

To determine the antioxidant effect of polyphenols, we determined nuclear-factor-erythroid 2–related factor 2 (Nrf2), heme oxidase-1 (HO-1), and NAD(P)H dehydrogenase [quinone] 1 (NQO1) levels in the colon tissue. The IHC staining results for Nrf2, HO-1, and NQO1 are presented in Figure 6. The expression levels of Nrf2 (Figure 7A,D) and HO-1 (Figure 7B,E) in model group was decreased compared with the control group. However, Nrf2 and HO-1 levels were significantly increased in the treatment groups compared with the model group. Lastly, NQO1 levels in the TA and EA groups were also significantly increased compared with model group (Figure 7C,F).

#### 2.2.6. Effect of Polyphenols on the Gut Microbiota

**Figure 8:**
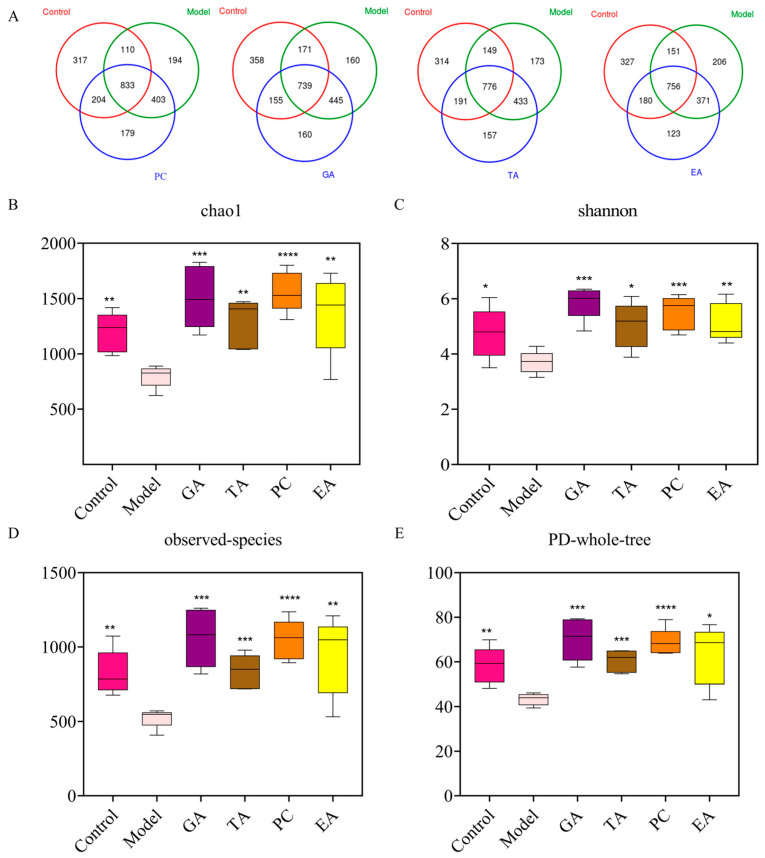
Effect of polyphenols administration on gut microbiota in dextran-sulfate-sodium (DSS)-induced colitis in mice (*n* = 3 per group). (**A**) Venn diagram showing the overlap of OTUs identified in the gut microbiota among the polyphenol groups and the control group and model group; (**B**–**E**) alpha diversity indicated by the Chao, Shannon, observed species, and PD-whole-tree indexes; gallic acid (GA); proanthocyanidins (PC); ellagic acid (EA); and tannic acid (TA); * represents *p* < 0.05; ** represents *p* < 0.005; *** represents *p* < 0.0005; **** represents *p* < 0.0001.

**Figure 9:**
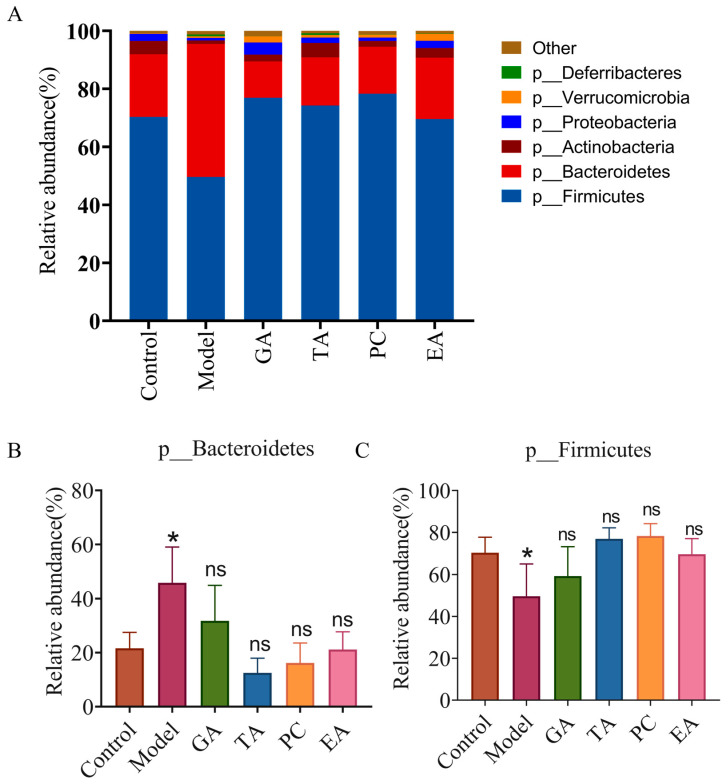
Effects of polyphenols on the composition of gut microbiota at the phylum level (*n* = 3 per group). (**A**) Histogram of the relative abundance of the dominant bacterial phyla; (**B**,**C**) polyphenols reversed the effect on the Bacteroidetes and Firmicutes; gallic acid (GA); proanthocyanidins (PC); ellagic acid (EA) and tannic acid (TA); ^ns^—no significance; * represents *p* < 0.05.

**Figure 10:**
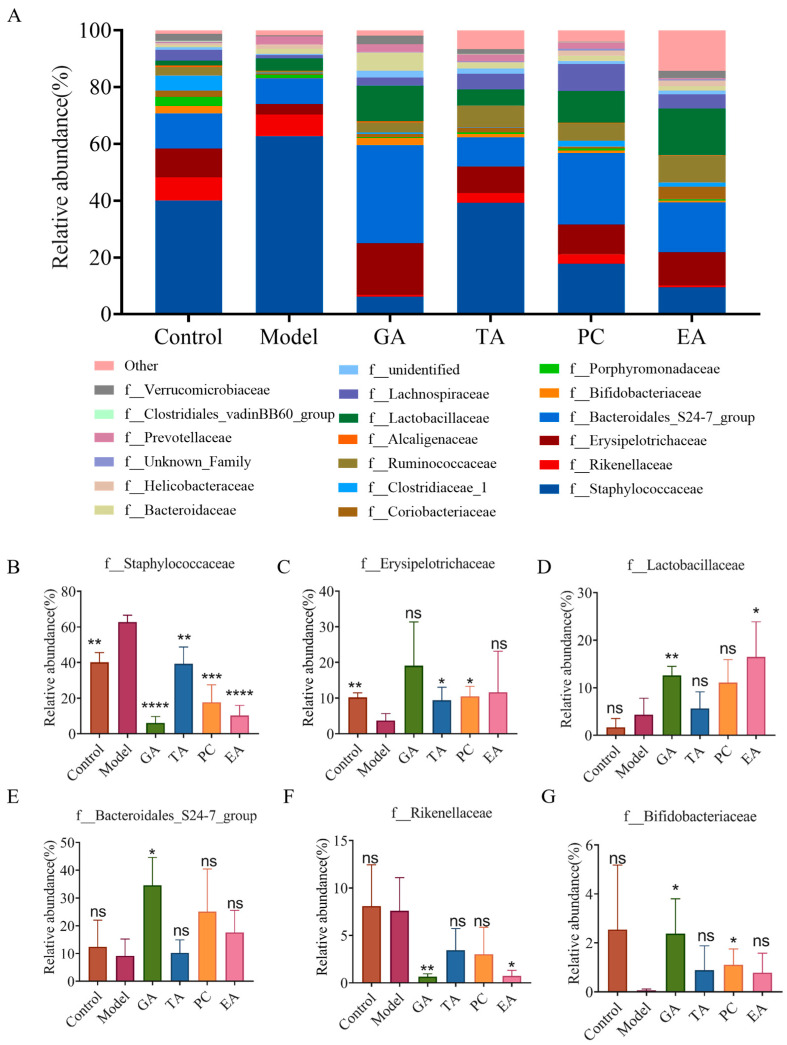
Effects of polyphenols on the composition of gut microbiota at the family level (*n* = 3 per group). (**A**) Histogram of the relative abundance of the dominant bacterial family. (**B**–**G**) Polyphenols modulated specific bacteria at the family level; gallic acid (GA); proanthocyanidins (PC); ellagic acid (EA); tannic acid (TA); ^ns^—no significance; * represents *p* < 0.05; ** represents *p* < 0.005; *** represents *p* < 0.0005; **** represents *p* < 0.0001.

**Figure 11:**
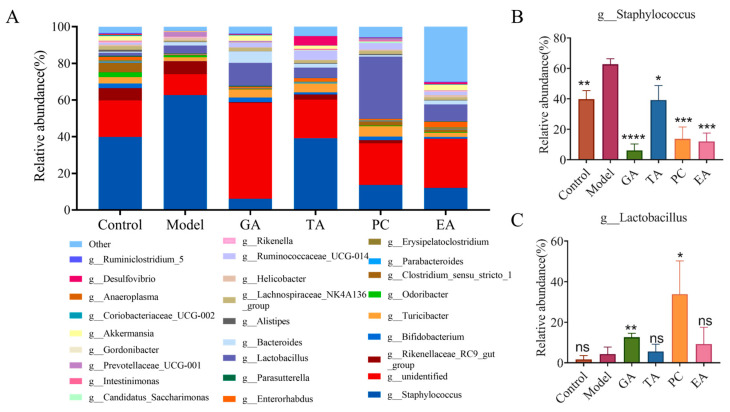
Effects of polyphenols on the composition of gut microbiota at the genus level (*n* = 3 per group). (**A**) Histogram of the relative abundance of the cumulative abundance of genera (**B**) and (**C**) polyphenols regulated specific bacterial genera. Gallic acid (GA); proanthocyanidins (PC); ellagic acid (EA); tannic acid (TA); ^ns^—no significance; * represents *p* < 0.05; ** represents *p* < 0.005; *** represents *p* < 0.0005; **** represents *p* < 0.0001.

Changes in the intestinal microbiome are one of the characteristics of UC pathogenesis [32]. To further evaluate the protective effect of polyphenols on UC, we elucidated microbiota changes induced by the polyphenols via 16s rRNA sequencing. The Venn diagram shows three overlapping sets of operational taxonomic units (OTUs), suggesting that there is a significant difference in OTUS between the DSS group and the other two groups (Figure 8A). In addition, compared with the control group, the alpha diversity of the microbiota in the model group was decreased, as indicated by a decrease in Chao index, Shannon, observed species, and PD whole-tree indices. However, the addition of polyphenols reversed the decline in the bacterial community richness index (Figure 8B–E). The histogram in Figure 9 reveals the species and relative abundance of the intestinal microbiota at the phylum level. Compared with the control group, the abundance of Firmicutes decreased, and that of Bacteroidetes increased in the model group. After polyphenol treatment, Bacteroidetes decreased significantly and Firmicutes increased significantly. At the family level, the relative abundance of Staphylococcaceae was increased and that of Erysipelotrichaceae was decreased in the model group compared to that in the control group. The use of polyphenols reversed the increase of Staphylococcaceae, and TA and PC reversed the decrease of Erysipelotrichaceae. In addition, treatment with GA and EA increased the relative abundance of Lactobacillaceae, and treatment with GA increased the relative abundance of Bacteroidales. However, treatment with GA and EA decreased the relative abundance of Rikenellaceae, and treatment with GA and PC increased the relative abundance of Bifldobacteriaceae (Figure 10). At the genus level, the relative abundance of *Staphylococcus* was markedly increased in the model group compared with the control group. Treatment with polyphenols decreased the relative abundances of *Staphylococcus* compared with the model group. Interestingly, treatment with GA and PC increased the relative abundances of *Lactobacillus* (Figure 11).

#### 2.2.7. Correlation Analysis

**Figure 12:**
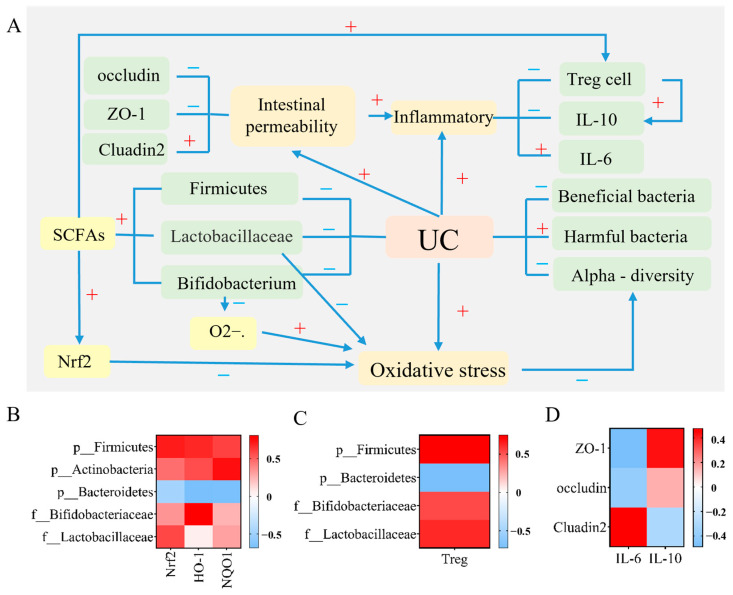
(**A**) The pathogenesis of UC and its correlation; heat map of (**B**) depicting the correlational analysis between antioxidant markers and the intestinal microbiota, (**C**) depicting the correlational analysis between Treg cells and the microbiota, and (**D**) depicting the correlational analysis among inflammatory cytokines, immune cells, and tight junction proteins; ulcerative colitis (UC); short-chain fatty acid (SCFA); nuclear-factor-erythroid 2–related factor 2 (Nrf2); heme oxidase-1 (HO-1); NAD(P)H dehydrogenase [quinone] 1 (NQO1); regulatory T (Treg); interleukin-6 (IL-6); and interleukin-10 (IL-10).

The correlation between intestinal microbiota and Nrf2, HO-1, NQO1, intestinal microbiota and Treg cells, inflammatory factors (IL-6, IL-10), immune cell (Treg cells), and tight junction proteins was analyzed. As shown in Figure 12A, UC pathogenesis includes changes in inflammation levels, breakdown of the intestinal barrier, and imbalance in the intestinal microbiome. The indicators involved in the three mechanisms were correlated with each other. Firmicutes and Proteobacteria were positively correlated with Nrf2, HO-1, and NQO1 levels; Bacteroidetes were negatively correlated with Nrf2, HO-1, and NQO1 levels; Bifidobacterium and Lactobacillaceae were positively correlated with Nrf2 and HO-1 levels (Figure 12B). Furthermore, Firmicutes, Bifidobacterium, and *Lactobacillus* were positively correlated with Treg cells, whereas Bacteroidetes were negatively correlated with Treg cells (Figure 12C). The proinflammatory, cytokine IL-6, was negatively correlated with ZO-1 and occludin and positively correlated with claudin-2, whereas the anti-inflammatory cytokine IL-10 and Treg cells were positively correlated with ZO-1 and occludin and negatively correlated with Cluadin2 (Figure 12D).

#### 2.2.8. Polyphenols Relieve Colitis-Associated Splenomegaly

**Figure 13:**
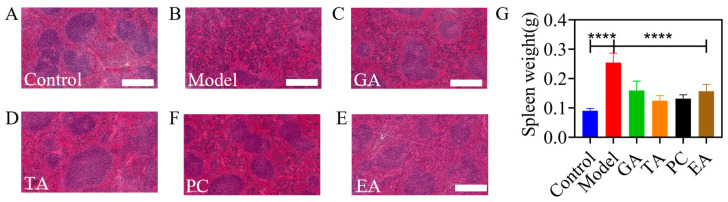
(**A**–**F**) Hematoxylin-eosin staining results of the spleen (×200), scale bar = 500 μm; (**G**) changes of spleen weight in mice; gallic acid (GA); proanthocyanidins (PC); ellagic acid (EA); tannic acid (TA); **** represents *p* < 0.0001.

We observed that the polyphenols significantly prevented the increase in spleen weight when compared with the model group (Figure 13G). H&E staining results of the spleen are presented in Figure 13. The mice in the control group had more white pulp, with the central artery located in the white pulp, and the marginal zone at the junction of the white and red pulp was obvious. In mice in the model group, the red pulp continued to extend, and the spleen was almost entirely composed of hyperplastic hematopoietic tissue and dominated by red tissue. The white pulp was significantly reduced. Furthermore, the marginal zone at the junction of white pulp and red pulp was inconspicuous. In GA, PC and TA group, there was a small degree of hematopoietic tissue hyperplasia, and the marginal zone at the junction of white pulp and red pulp was obvious. In EA group, there was no red pulp expansion and the marginal zone at the junction of white pulp and red pulp was obvious. The histological structure of the spleen in the EA group was similar to that of the spleen in the control group.

#### 2.2.9. In Vivo Safety Evaluation

**Figure 14:**
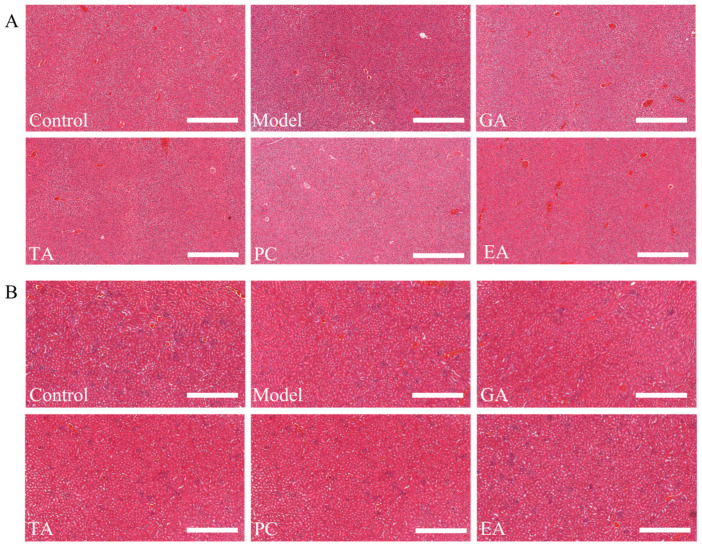
(**A**) H&E staining results of the liver and (**B**) the kidney (×200) (*n* = 6 per group), scale bar = 500 μm; gallic acid (GA); proanthocyanidins (PC); ellagic acid (EA); tannic acid (TA).

H&E staining results of the liver and kidney are shown in Figure 14. Histology examination found no damaging effects of polyphenol administration on the kidney and liver.

## 3. Discussion

In this study, we analyzed the antioxidant activity and the antioxidant structural relationship of the four polyphenols, and studied their alleviating effects on UC. Studies have shown that polyphenols inhibit the production of free radicals by inhibiting the activities of the oxidoreductive enzymes and/or chelating to prevent the production of metals via free radicals [33,34,35]. The phenol group accepts electrons to form phenoxy, interrupting the chain oxidation reaction. Conjugated aromatic systems can delocalize unpaired electrons. The antioxidant capacity of polyphenols is related to the number of hydroxyl groups and characteristic structural groups such as the o-phenol group, in which o-phenol can make free radicals form with higher stability [36]. In the present study, TA had twenty-five phenolic hydroxyl groups, including five catechol groups and five pyrogallol groups; PC had eight phenolic hydroxyl groups, including two catechol groups; GA had three phenol hydroxyl groups, which are the pyrogallol group; and EA had four phenol hydroxyl groups, with two catechol groups. The antioxidant activity of polyphenols was measured via determining the DPPH radical scavenging activity, ABTS radical scavenging activity, and total antioxidant capacity. All four polyphenols exhibited good antioxidant capacity. At lower concentrations, TA still exhibited the strongest antioxidant properties, owing to the highest number of phenol hydroxyl and o-phenol groups, followed by PC. PC exhibited stronger ABTS free radical scavenging ability than GA, and EA ranks last. Among them, the scavenging ability of GA was better than that of EA; this suggests that when the number of phenol hydroxyl groups is similar, compounds with more pyrogallol groups exhibit better antioxidant activity than those with catechol groups [36].

UC is one of the most common gastrointestinal disorders characterized by chronic, recurrent inflammation that causes damage to the colon mucosa [31]. It can occur at all ages, leading to lifetime morbidity and even death [37,38]. Several studies have shown that polyphenols help relieve colitis [39,40]. In the present study, we elucidated the effects of four polyphenols in a DSS-induced UC mouse model. We observed that the polyphenols significantly improved colitis based on weight recovery, colon length recovery, and decreased DAI. In addition, several potential mechanisms were described, including ameliorating intestinal barrier damage, regulating inflammatory responses and oxidative stress, and reshaping of the intestinal microbiota of mice.

TJs include the transmembrane proteins, occludin and claudin family members, and proteins such as claudin-2 and ZO-1 [23,41,42]. ZO-1, a major TJ protein, is associated with epithelial integrity and can be used as a marker of the intestinal barrier. Occludin plays an important role in TJ stability and barrier function [42,43]. Claudin-2 is a typical pore-forming claudin that forms active gated channels, with selectivity for water and small cations. Studies have reported that claudin-2 levels are increased in IBD and that claudin-2 degradation can enhance the intestinal barrier of TJs [41,44,45]. In the present study, we demonstrated that occludin and ZO-1 levels were decreased and claudin-2 levels were increased after DSS administration and that the four polyphenols can increase ZO-1 and occludin levels, whereas GA, PC, and TA can decrease claudin-2 levels; these results indicate that the four polyphenols can relieve UC by repairing the intestinal barrier.

Increasing evidence suggests that proinflammatory cytokines are critical in UC pathogenesis. Elevated IL-6, a proinflammatory cytokine, levels are a major feature of UC [46,47,48]. In the present study, proinflammatory cytokine levels were increased in the model group. However, GA, PC, and EA treatment significantly decreased proinflammatory cytokine secretion. A study reported that the anti-inflammatory cytokine IL-10 can improve intestinal damage caused by UC [49]. Consistent with these findings, we observed that IL-10 levels were increased in the PC and EA groups. These results suggest that polyphenols play important roles in regulating intestinal inflammation in DSS-induced UC. In addition, Treg cells can play an anti-inflammatory role in various immune diseases by secreting anti-inflammatory cytokines, including IL-10, and inhibiting immune cell activity [50,51]. In the present study, we observed that GA, PC, and EA could significantly improve the activation level of Treg cells; this was consistent with the tendency of polyphenols to regulate inflammatory cytokines.

Nrf2 is an important anti-inflammatory and antioxidant signaling pathway molecule that is closely associated with UC occurrence. It plays an antioxidant role by promoting the expression of the downstream molecules HO-1 and NQO-1 [31,52]. We demonstrated that polyphenols can increase the protein levels of HO-1 and Nrf2 in UC mice. Furthermore, EA and TA can increase NQO1 protein levels. These results suggest that polyphenols play an anti-inflammatory role by enhancing antioxidant activity.

Many studies have reported that intestinal microbial environment disorders play an important role in UC pathogenesis. Compared with healthy individuals, intestinal microbial disturbances in UC patients have demonstrated reduced diversity and richness, and changes in the intestinal microbiota composition [53,54]. Consistent with the findings of a previous study [2,23,55], we found that Firmicutes, Bacteroidetes, Actinobacteria, and Proteobacteria are the dominant phyla, and their compositions changed. After polyphenol intervention, these changes were reversed to varying degrees. *Staphylococcus aureus* is a kind of intestinal pathogenic bacteria [7,23]. Polyphenol intervention reduced the relative abundance of *Staphylococcus aureus*. Simultaneously, treatment with polyphenols decreased the relative abundance of *Staphylococcus* at the genus level. *Lactobacillus* and *Bifidobacteria* secrete inflammatory inhibitors by downregulating NF-κB-dependent gene expression, IL-8 secretion, and macrophage chemokine levels. Furthermore, they downregulate effector T-cell-mediated inflammatory responses but upregulate the expression of anti-inflammatory Treg cells in mice [7,56]. Some byproducts of short-chain fatty acids (SCFAs) exhibit anti-inflammatory effects and may enhance the intestinal barrier [7]. *Bifidobacteria* is an SCFA-producing bacteria [56]. GA and PC significantly increased the relative abundance of *Lactobacillus* and Bifidobacteriaceae. Erysipelotrichaceae is a potential probiotic [57], and a study has shown that it is a butyrate producing bacteria, which is a SCFAs [58]. In the present study, TA and PC significantly increased the relative abundance of Erysipelotrichaceae. A study clarified that the Bacteroidales S24-7 group can reduce pro-inflammatory activity and play an immunomodulatory role [59]. In our study, GA increased the enrichment of the Bacteroidales S24-7 group. Based on these results, polyphenols can relieve UC by regulating the imbalance of microbiota, increasing the abundance of beneficial bacteria, and reducing the abundance of harmful bacteria in the intestine.

In addition, studies have shown that gut microbes influence the gut’s antioxidant response, which is essential for gut health [60,61]. Oxidative stress during the inflammatory response can reduce intestinal microbial diversity [62], whereas an imbalance in intestinal microbiota can increase oxidative damage and decrease antioxidant capacity by destroying the intestinal barrier [63]. Studies have reported that probiotics play beneficial roles in regulating oxidative stress responses. For example, lactic acid bacteria can improve the antioxidant capacity of the body, Bifidobacteriaceae can remove free radicals and superoxide anions, and enhance the activity of antioxidant enzymes [64]. Meanwhile, SCFAs can activate the Nrf2 pathway [60]; Bifidobacteriaceae is an SCFA-producing bacteria (Figure 11A). Intestinal microbiota analysis revealed that GA, PC, and EA can improve the relative abundance of Lactobacillaceae and Bifidobacteriaceae. In addition, we determined the correlation between the abundance of intestinal microbiota and the expression of oxidative stress biomarkers. At the phylum level, when the relationship between oxidative stress biomarkers and intestinal microbial abundance was analyzed, Nrf2, HO-1, and NQO1 levels were positively correlated with the relative abundances of Firmicutes and Actinobacteria and negatively correlated with the relative abundance of Bacteroides. At the family level, when the relationship between oxidative stress biomarkers and intestinal microbial abundance was analyzed, Nrf2, HO-1, and NQO1 levels were positively correlated with Lactobacillaceae and Bifidobacteriaceae (Figure 11B). The byproducts of SCFAs are butyrate, propionic acid, acetate, etc. Imbalance in the intestinal microbiota and an increase in intestinal inflammatory cells in patients with IBD are associated with a decrease in SCFA levels, which can regulate intestinal immunity and play an anti-inflammatory role by activating the differentiation and expansion of Treg cells [65]. Studies have reported that some bacteria in Firmicutes can produce butyrate; lactic acid bacteria have butyrate-producing capacity; and Bifidobacterium can produce acetic acid, propionic acid, butyric acid, lactic acid, and other SCFAs [66]. In the present study, correlation analysis between the intestinal microbiota and Treg cells revealed that Firmicutes, Lactobacillus, and Bifidobacterium were positively correlated with Treg cells (Figure 11C). Barrier integrity is mostly due to the normal functioning of the TJs between epithelial cells. TJs comprise transmembrane proteins that regulate intestinal barrier permeability by controlling the diffusion of water, ions, and nutrients while limiting pathogen entry. The intestinal barrier dysfunction can exacerbate inflammation throughout the body [67]. In the present study, we determined the correlation between inflammatory cytokine levels and intestinal TJ protein levels. We observed that ZO-1 and occludin, which exhibit protective effects on intestinal permeability, were negatively correlated with proinflammatory cytokines and positively correlated with anti-inflammatory cytokines and Treg cells. Claudin-2 protein levels, which increase intestinal permeability, were positively correlated with proinflammatory cytokines and negatively correlated with anti-inflammatory cytokines and Treg cells (Figure 11D).

In summary, four plant polyphenols were shown to alleviate UC through multiple mechanisms. Many plants which are traditionally used in China contain these polyphenols. *Lycium ruthenicum* Murray (LR), for example, is a kind of medicinal and edible plant widely distributed in the salinized desert of Northwestern China. LR has been widely used in food, medicine, and other fields, as is of great economic value [68]. Studies have found that LR contains the four polyphenols [69,70,71]. Hence, the potential therapeutic mechanism of LR for ulcerative colitis can be explored.

## 4. Materials and Methods

### 4.1. Materials

TA and GA were purchased from Sigma Aldrich Chemie (St. Louis, Missouri, USA). EA and PC were purchased from Xi’an Green Biotechnique Co., Ltd. (Xi’an, China).

### 4.2. In Vitro Antioxidant Assay

#### 4.2.1. DPPH·-Radical-Scavenging Assay

The antioxidant activity of the four polyphenols were evaluated by measuring their stable-DPPH·-free-radical-scavenging ability [72,73]. A series of polyphenol compound solutions were prepared, (10, 5, 2.5, 1.25, 0.625, 0.3125, 0.1563, 0.0781, 0.625, 0.0391, and 0.0195 mM). Then, 0.006 g of DPPH· powder (Shanghai Macklin Biochemical Co., Ltd., Shanghai, China) was dissolved in 50 mL of anhydrous ethanol and stored in the dark. The samples and DPPH alcohol solution were mixed in 96-well plates and incubated for 30 min at 25 °C in the dark. The absorbance was measured at 517 nm using an enzyme-labeled instrument. DPPH· degradation was calculated using the equation from the previous study [74].

#### 4.2.2. ABTS-Scavenging-Assay

The antioxidant activity of the four polyphenols was evaluated by measuring their scavenging ability on ABTS free radicals and preparing polyphenolic compound solutions of a series of concentrations, followed by the determination of the ABTS scavenging rates by using an ABTS free radical clear-ability assay Kit (Beijing Solarbio Science & Technology Co., Ltd., Beijing, China).

#### 4.2.3. Total Antioxidant Capacity

The antioxidant activity of the four polyphenols was evaluated by calculating the total antioxidant capacity. The polyphenolic compounds solutions were prepared in a series of concentrations. The sample solution was added to the 96-well plate, to which the FRAP working solution (Beyotime, Shanghai China) was added and the solution was incubated at 37 °C for 3 min. The absorbance was measured at 593 nm wavelength by using an enzyme-labeled instrument. The total antioxidant capacity is expressed as the concentration of the FeSO_4_ standard solution.

### 4.3. In Vivo Assessment of the Restorative Effects of UC

#### 4.3.1. Experimental Animals

Forty-two male C57BL/6 mice (9–10 weeks of age) were purchased from SPF (Beijing, China) Biotechnology Co., Ltd. The mice were accommodated for 1 week. All the plans were approved by the Animal experiment Committee of Peking Union Medical College.

#### 4.3.2. Animal Experimental Design

Mice were randomly divided into a control group (*n* = 6) and a DSS group (*n* = 36). The control group received normal water and the DSS group received 3% DSS (35~50 kD, MP Biologicals, Solon, OH, USA) solution (*w*/*v*) to induce acute UC. After 7 days, the mice in the DSS group were divided into the model group and GA, TA, PC, and EA groups, with an equal number of mice in each group. The control and model groups were administered normal water via gavage every morning for 6 days. Conversely, the treatment groups were administered a 100 mg/kg polyphenol solution.

The weight of the mice was recorded daily throughout the experiment. DAI combined weight loss score, stool diarrhea score, and fecal blood levels were used to evaluate UC severity [42,75]. On the 14th day, the mice were sacrificed, and the length of their colons was photographed and measured. Then, 5 mm of the colons was removed and cleaned with normal saline and fixed with 4% paraformaldehyde for subsequent sections and staining. The remaining colon tissues were washed with normal saline and quickly frozen with liquid nitrogen for subsequent analysis. The spleen tissue was flushed with physiological saline and then weighed. The spleen, liver, and kidney tissues were fixed in 4% paraformaldehyde for subsequent sectioning and staining. Fecal samples from mice were collected and stored at −80 °C for subsequent analysis. Serum was obtained via centrifugation (1200× *g*, 4 °C, 15 min) for measuring cytokine levels. The concentrations of IL-6 and IL-10 were measured by using an enzyme-linked immunoassay kit (Thermo Fisher Scientific, Massachusetts, MA, USA).

#### 4.3.3. Histological Analysis

Colon tissue sections were fixed with paraformaldehyde and stained with H&E for morphological measurements. All sections were analyzed and photographed under a microscope (Leica, Wetzlar, Germany).

#### 4.3.4. Immunohistochemistry

The colon tissue sections fixed by paraformaldehyde were dewaxed, rehydrated, extracted antigens, and stained, and then stained with claudin-2, ZO-1, occluding, and FOXP3 antibodies (Abcam, Cambridge, UK). All sections were analyzed and photographed under a microscope (Leica, Wetzlar, Germany).

#### 4.3.5. Impact on Gut Microbiota

The collected fecal samples were analyzed by 16S rRNA sequencing at Beijing Yike Baide Technology Co., Ltd. (Beijing, China) for intestinal microbiota analysis. Samples processing includes whole genome DNA extraction, PCR amplification, amplicon quantization, and high throughput sequencing. The sequencing results were analyzed on the company’s interactive platform.

### 4.4. Statistical Analysis

GraphPad Prism 8.0.2 and origin 2021 software were used for mapping and statistical analysis, and the results were expressed by mean ± SD. One-way ANOVA and Tukey’s test were used to analyze the differences between groups.

## 5. Conclusions

We elucidated the antioxidant effects of four polyphenols in plants and on DSS-induced colitis. Our study results indicate that the antioxidant capacity of polyphenols is associated with the number of phenolic hydroxyl groups and the structure of o-phenol groups. The studied polyphenols can alleviate UC, including decreasing DAI and improving histological alteration. The mechanism involves regulation of inflammatory factors, intestinal barrier repair, regulation of oxidative stress, and regulation of intestinal microbiota. The functions of several mechanisms are interrelated and influence each other. Moreover, the polyphenols have good biosafety. The results suggest that polyphenols play an important role in UC remission, may provide a basis for the development of products containing plant polyphenols in the prevention and treatment of colitis.

## Data Availability

Data are contained within the article.

## References

[1] Liu S., Cao Y., Ma L., Sun J., Ramos-Mucci L., Ma Y., Yang X., Zhu Z., Zhang J., Xiao B. (2022). Oral antimicrobial peptide-EGCG nanomedicines for synergistic treatment of ulcerative colitis. J. Control. Release.

[2] Kitabatake M., Matsumura Y., Ouji-Sageshima N., Nishioka T., Hara A., Kayano S.I., Ito T. (2021). Persimmon-derived tannin ameliorates the pathogenesis of ulcerative colitis in a murine model through inhibition of the inflammatory response and alteration of microbiota. Sci. Rep..

[3] Zhang Z., Shen P., Xie W., Cao H., Liu J., Cao Y., Zhang N. (2019). Pingwei San ameliorates dextran sulfate sodium-induced chronic colitis in mice. J. Ethnopharmacol..

[4] Danese S., Roda G., Peyrin-Biroulet L. (2020). Evolving therapeutic goals in ulcerative colitis: Towards disease clearance. Nat. Rev. Gastroenterol. Hepatol..

[5] Chao L., Lin J., Zhou J., Du H., Chen X., Liu M., Qu Q., Lv W., Guo S. (2022). Polyphenol Rich *Forsythia suspensa* Extract Alleviates DSS-Induced Ulcerative Colitis in Mice through the Nrf2-NLRP3 Pathway. Antioxidants.

[6] Wu X., Xu N., Ye Z., Zhao Q., Liu J., Li J., Wu M., Zheng Y., Li X., Li W. (2022). Polysaccharide from *Scutellaria barbata* D. Don attenuates inflammatory response and microbial dysbiosis in ulcerative colitis mice. Int. J. Biol. Macromol..

[7] Singh R.K., Chang H.W., Yan D., Lee K.M., Ucmak D., Wong K., Abrouk M., Farahnik B., Nakamura M., Zhu T.H. (2017). Influence of diet on the gut microbiome and implications for human health. J. Transl. Med..

[8] Darnaud M., Santos A.D., Gonzalez P., Augui S., Lacoste C., Desterke C., De Hertogh G., Valentino E., Braun E., Zheng J. (2018). Enteric Delivery of Regenerating Family Member 3 alpha Alters the Intestinal Microbiota and Controls Inflammation in Mice with Colitis. Gastroenterology.

[9] Li X., Wei X., Sun Y., Du J., Li X. (2019). High-fat diet promotes experimental colitis by inducing oxidative stress in the colon. Am. J. Physiol..

[10] Xiao B., Zhang Z., Viennois E., Kang Y., Zhang M., Han M.K., Chen J., Merlin D. (2016). Combination Therapy for Ulcerative Colitis: Orally Targeted Nanoparticles Prevent Mucosal Damage and Relieve Inflammation. Theranostics.

[11] Qiu P., Ishimoto T., Fu L., Zhang J., Zhang Z., Liu Y. (2022). The Gut Microbiota in Inflammatory Bowel Disease. Front. Cell Infect. Microbiol..

[12] Lee Y., Sugihara K., Gillilland M.G., Jon S., Kamada N., Moon J.J. (2020). Hyaluronic acid-bilirubin nanomedicine for targeted modulation of dysregulated intestinal barrier, microbiome and immune responses in colitis. Nat. Mater..

[13] Nighot P., Al-Sadi R., Rawat M., Guo S., Watterson D.M., Ma T. (2015). Matrix metalloproteinase 9-induced increase in intestinal epithelial tight junction permeability contributes to the severity of experimental DSS colitis. Am. J. Physiol. Gastrointest. Liver Physiol..

[14] Yi J., Liu X. (2019). Advances in intestinal barrier function in inflammatory bowel disease. Chin. J. Inflamm. Bowel Dis..

[15] Ozdal T., Sela D.A., Xiao J., Boyacioglu D., Chen F., Capanoglu E. (2016). The Reciprocal Interactions between Polyphenols and Gut Microbiota and Effects on Bioaccessibility. Nutrients.

[16] Wan M.L.Y., Ling K.H., El-Nezami H., Wang M.F. (2019). Influence of functional food components on gut health. Crit. Rev. Food Sci. Nutr..

[17] Moco S., Martin F.P., Rezzi S. (2012). Metabolomics view on gut microbiome modulation by polyphenol-rich foods. J. Proteome Res..

[18] Marin L., Miguelez E.M., Villar C.J., Lombo F. (2015). Bioavailability of dietary polyphenols and gut microbiota metabolism: Antimicrobial properties. Biomed. Res. Int..

[19] Wu Z., Huang S., Li T., Li N., Han D., Zhang B., Xu Z.Z., Zhang S., Pang J., Wang S. (2021). Gut microbiota from green tea polyphenol-dosed mice improves intestinal epithelial homeostasis and ameliorates experimental colitis. Microbiome.

[20] Sheng K., Zhang G., Sun M., He S., Kong X., Wang J., Zhu F., Zha X., Wang Y. (2020). Grape seed proanthocyanidin extract ameliorates dextran sulfate sodium-induced colitis through intestinal barrier improvement, oxidative stress reduction, and inflammatory cytokines and gut microbiota modulation. Food Funct..

[21] Derosa G., Maffioli P., Sahebkar A. (2016). Ellagic Acid and Its Role in Chronic Diseases. Adv. Exp. Med. Biol..

[22] Shakeri A., Zirak M.R., Sahebkar A. (2018). Ellagic Acid: A Logical Lead for Drug Development?. Curr. Pharm. Des..

[23] Singh B., Singh J.P., Kaur A., Singh N. (2017). Phenolic composition and antioxidant potential of grain legume seeds: A review. Food Res. Int..

[24] Bing X., Xuelei L., Wanwei D., Linlang L., Keyan C. (2017). EGCG Maintains Th1/Th2 Balance and Mitigates Ulcerative Colitis Induced by Dextran Sulfate Sodium through TLR4/MyD88/NF-kappaB Signaling Pathway in Rats. Can. J. Gastroenterol. Hepatol..

[25] Leung L.K., Su Y., Chen R., Zhang Z., Huang Y., Chen Z.Y. (2001). Theaflavins in black tea and catechins in green tea are equally effective antioxidants. J. Nutr..

[26] Zheng B., He Y., Zhang P., Huo Y.X., Yin Y. (2022). Polyphenol Utilization Proteins in the Human Gut Microbiome. Appl. Environ. Microbiol..

[27] Marin M., Giner R.M., Rios J.L., Recio M.C. (2013). Intestinal anti-inflammatory activity of ellagic acid in the acute and chronic dextrane sulfate sodium models of mice colitis. J. Ethnopharmacol..

[28] Liu Z.G., Dang J., Wang Q.L., Yu M., Jiang L., Mei L., Shao Y., Tao Y. (2013). Optimization of polysaccharides from *Lycium ruthenicum* fruit using RSM and its an-ti-oxidant activity. Int. J. Biol. Macromol..

[29] Peng Q., Liu H., Shi S., Li M. (2014). *Lycium ruthenicum* polysaccharide attenuates inflammation through inhibiting TLR4/NFκB signaling pathway. Int. J. Biol. Macromol..

[30] Song J.L., Gao Y., Xu J. (2014). Protective effects of methanolic extract from fruits of *Lycium ruthenicum* Murr. on 2,2′-azobis(2-amidinopropane) dihydrochloride-induced oxidative stress in LLC-PK1 cells. Pharmacogn. Mag..

[31] Qiu S., Li P., Zhao H., Li X. (2020). Maresin 1 alleviates dextran sulfate sodium-induced ulcerative colitis by regulating NRF2 and TLR4/NF-kB signaling pathway. Int. Immunopharmacol..

[32] Limon J.J., Tang J., Li D., Wolf A.J., Michelsen K.S., Funari V., Gargus M., Nguyen C., Sharma P., Maymi V.I. (2019). Malassezia Is Associated with Crohn’s Disease and Exacerbates Colitis in Mouse Models. Cell Host Microbe.

[33] Dini I., Grumetto L. (2022). Recent Advances in Natural Polyphenol Research. Molecules.

[34] Amić A., Marković Z., Marković J.M.D., Jeremić S., Lučić B., Amić D. (2016). Free radical scavenging and COX-2 inhibition by simple colon metabolites of polyphenols: A theoretical approach. Comput. Biol. Chem..

[35] Cheng Y.C., Sheen J.M., Hu W.L., Hung Y.C. (2017). Polyphenols and Oxidative Stress in Atherosclerosis-Related Ischemic Heart Disease and Stroke. Oxid. Med. Cell Longev..

[36] Musial C., Kuban-Jankowska A., Gorska-Ponikowska M. (2020). Beneficial Properties of Green Tea Catechins. Int. J. Mol. Sci..

[37] Michael B., Oscar F.V., Robert S., Andy B., Sheena C. (2015). Quality of Methods Reporting in Animal Models of Colitis. Inflamm. Bowel Dis..

[38] Li H., Fan C., Feng C., Wu Y., Lu H., He P., Yang X., Zhu F., Qi Q., Gao Y. (2019). Inhibition of phosphodiesterase-4 attenuates murine ulcerative colitis through interference with mucosal immunity. Br. J. Pharmacol..

[39] Yang R., Shan S., An N., Liu F., Cui K., Shi J., Li H., Li Z. (2022). Polyphenols from foxtail millet bran ameliorate DSS-induced colitis by remodeling gut microbiome. Front. Nutr..

[40] Guo F., Tsao R., Li C., Wang X., Zhang H., Jiang L., Sun Y., Xiong H. (2021). Green Pea (*Pisum sativum* L.) Hull Polyphenol Extracts Ameliorate DSS-Induced Colitis through Keap1/Nrf2 Pathway and Gut Microbiota Modulation. Foods.

[41] Guo Y., Xu C., Gong R., Hu T., Zhang X., Xie X., Chi J., Li H., Xia X., Liu X. (2022). Exosomal CagA from *Helicobacter pylori* aggravates intestinal epithelium barrier dysfunction in chronic colitis by facilitating Claudin-2 expression. Gut. Pathog..

[42] Peng Y., Yan Y., Wan P., Chen D., Ding Y., Ran L., Mi J., Lu L., Zhang Z., Li X. (2019). Gut microbiota modulation and anti-inflammatory properties of anthocyanins from the fruits of *Lycium ruthenicum* Murray in dextran sodium sulfate-induced colitis in mice. Free Radic. Biol. Med..

[43] Wu H., Chen Q.Y., Wang W.Z., Chu S., Liu X.X., Liu Y.J., Tan C., Zhu F., Deng S.J., Dong Y.L. (2021). Compound sophorae decoction enhances intestinal barrier function of dextran sodium sulfate induced colitis via regulating notch signaling pathway in mice. Biomed. Pharmacother..

[44] Nighot P.K., Hu C.A., Ma T.Y. (2015). Autophagy enhances intestinal epithelial tight junction barrier function by targeting claudin-2 protein degradation. J. Biol. Chem..

[45] Raju P., Shashikanth N., Tsai P.Y., Pongkorpsakol P., Chanez-Paredes S., Steinhagen P.R., Kuo W.T., Singh G., Tsukita S., Turner J.R. (2020). Inactivation of paracellular cation-selective claudin-2 channels attenuates immune-mediated experimental colitis in mice. J. Clin. Investig..

[46] Li X., Lv H., Shi F., Song J., Zhang Z. (2022). The potential therapeutic effects of hydroxypropyl cellulose on acute murine colitis induced by DSS. Carbohydr. Polym..

[47] Kim J.J., Shajib M.S., Manocha M.M., Khan W.I. (2012). Investigating intestinal inflammation in DSS-induced model of IBD. J. Vis. Exp..

[48] Strober W., Fuss I.J. (2011). Proinflammatory cytokines in the pathogenesis of inflammatory bowel diseases. Gastroenterology.

[49] Tu L., Gharibani P., Zhang N., Yin J., Chen J.D. (2020). Anti-inflammatory effects of sacral nerve stimulation: A novel spinal afferent and vagal efferent pathway. Am. J. Physiol. Gastrointest. Liver Physiol..

[50] Yan J.B., Luo M.M., Chen Z.Y., He B.H. (2020). The Function and Role of the Th17/Treg Cell Balance in Inflammatory Bowel Disease. J. Immunol. Res..

[51] Giganti G., Atif M., Mohseni Y., Mastronicola D., Grageda N., Povoleri G.A., Miyara M., Scotta C. (2021). Treg cell therapy: How cell heterogeneity can make the difference. Eur. J. Immunol..

[52] Zhou Y., Liu H., Song J., Cao L., Tang L., Qi C. (2018). Sinomenine alleviates dextran sulfate sodium-induced colitis via the Nrf2/NQO-1 signaling pathway. Mol. Med. Rep..

[53] Liu Y., Zhou M., Yang M., Jin C., Song Y., Chen J., Gao M., Ai Z., Su D. (2021). *Pulsatilla chinensis* Saponins Ameliorate Inflammation and DSS-Induced Ulcerative Colitis in Rats by Regulating the Composition and Diversity of Intestinal Flora. Front. Cell Infect. Microbiol..

[54] Pang B., Jin H., Liao N., Li J., Jiang C., Shi J. (2021). Vitamin A supplementation ameliorates ulcerative colitis in gut microbiota-dependent manner. Food Res. Int..

[55] Ni Y., Yang X., Zheng L., Wang Z., Wu L., Jiang J., Yang T., Ma L., Fu Z. (2019). Lactobacillus and Bifidobacterium Improves Physiological Function and Cognitive Ability in Aged Mice by the Regulation of Gut Microbiota. Mol. Nutr. Food. Res..

[56] Kawabata K., Yoshioka Y., Terao J. (2019). Role of Intestinal Microbiota in the Bioavailability and Physiological Functions of Dietary Polyphenols. Molecules.

[57] Li S., Zhuge A., Wang K., Lv L., Bian X., Yang L., Xia J., Jiang X., Wu W., Wang S. (2021). Ketogenic diet aggravates colitis, impairs intestinal barrier and alters gut microbiota and metabolism in DSS-induced mice. Food Funct..

[58] Liu S., Li E., Sun Z., Fu D., Duan G., Jiang M., Yu Y., Mei L., Yang P., Tang Y. (2019). Altered gut microbiota and short chain fatty acids in Chinese children with autism spectrum disorder. Sci. Rep..

[59] Lv J., Jia Y., Li J., Kuai W., Li Y., Guo F., Xu X., Zhao Z., Lv J., Li Z. (2019). Gegen Qinlian decoction enhances the effect of PD-1 blockade in colorectal cancer with microsatellite stability by remodelling the gut microbiota and the tumour microenvironment. Cell Death Dis..

[60] Ni Q., Zhang P., Li Q., Han Z. (2022). Oxidative Stress and Gut Microbiome in Inflammatory Skin Diseases. Front. Cell Dev. Biol..

[61] Ma J., Piao X., Mahfuz S., Long S., Wang J. (2021). The interaction among gut microbes, the intestinal barrier and short chain fatty acids. Anim. Nutr..

[62] Weiss G.A., Hennet T. (2017). Mechanisms and consequences of intestinal dysbiosis. Cell Mol. Life Sci..

[63] Tang J., Xu L., Zeng Y., Gong F. (2021). Effect of gut microbiota on LPS-induced acute lung injury by regulating the TLR4/NF-kB signaling pathway. Int. Immunopharmacol..

[64] Wang Y., Wu Y., Wang Y., Xu H., Mei X., Yu D., Wang Y., Li W. (2017). Antioxidant Properties of Probiotic Bacteria. Nutrients.

[65] Hybertson B.M., Gao B., Bose S.K., McCord J.M. (2011). Oxidative stress in health and disease: The therapeutic potential of Nrf2 activation. Mol. Aspects Med..

[66] Martin-Gallausiaux C., Marinelli L., Blottière H.M., Larraufie P., Lapaque N. (2021). SCFA: Mechanisms and functional im-portance in the gut. Proc. Nutr. Soc..

[67] Bach Knudsen K.E., Laerke H.N., Hedemann M.S., Nielsen T.S., Ingerslev A.K., Nielsen D.S.G., Theil P.K., Purup S., Hald S., Schioldan A.G. (2018). Impact of Diet-Modulated Butyrate Production on Intestinal Barrier Function and Inflammation. Nutrients.

[68] Li J., Yuan H., Ceng X.C., Han B., Shi D.H. (2007). Toxicological assessment of pigment of *Lycium ruthenicum* Murr. Food Sci..

[69] Lv X., Wang C., Cheng Y., Huang L., Wang Z. (2013). Isolation and structural characterization of a polysaccharide LRP4-A from *Lycium ruthenicum* Murr. Carbohydr. Res..

[70] Ou Y.F., Ji T.F., Su Y.L., Li J., Liu H. (2012). Chemcial constituents of the fruits of *Lycium ruthenicum*. Zhong Yao Cai.

[71] Zheng J., Ding C.X., Wang L.S., Lia G., Shia J., Li H., Wanga H., Suoa Y. (2011). Anthocyanins composition and antioxidant activity of wild *Lycium ruthenicum* Murr. from Qinghai-Tibet Plateau. Food Chem..

[72] Wang K., Wang Y., Lin S., Liu X., Yang S., Jones G.S. (2015). Analysis of DPPH inhibition and structure change of corn pep-tides treated by pulsed electric field technology. J. Food Sci. Technol..

[73] Rajakumari R., Volova T., Oluwafemi O.S., Kumar S.R., Thomas S., Kalarikkal N. (2020). Grape seed extract-soluplus dispersion and its antioxidant activity. Drug Dev. Ind. Pharm..

[74] Chen H., Zheng T., Wu C., Wang J., Ye F., Cui M., Sun S., Zhang Y., Li Y., Dong Z. (2022). A Shape-Adaptive Gallic Acid Driven Multifunctional Adhesive Hydrogel Loaded with Scolopin2 for Wound Repair. Pharmaceuticals.

[75] Stillie R.M., Stadnyk A.W. (2010). Role of TNF receptors, TNFR1 and TNFR2, in dextran sodium sulfate-induced colitis. Inflamm. Bowel Dis..

